# UNMET NEEDS OF CAREGIVERS OF HOSPITALIZED VETERANS LIVING WITH DEMENTIA: A QUALITATIVE STUDY

**DOI:** 10.1093/geroni/igad104.2979

**Published:** 2023-12-21

**Authors:** Molly Horstman, Crystal Guo, Mandi Sonnenfeld, Tracy Evans, Alan Stevens, Mark Kunik

**Affiliations:** Michael E. DeBakey VA Medical Center, Houston, Texas, United States; Baylor College of Medicine, Houston, Texas, United States; Michael E. DeBakey VA Medical Center, Houston, Texas, United States; Baylor College of Medicine, Houston, Texas, United States; Baylor Scott and White Health, Dallas, Texas, United States; Baylor College of Medicine, Houston, Texas, United States

## Abstract

Family caregivers are essential to safe care transitions for hospitalized adults living with dementia. We aimed to identify resource and training needs of dementia caregivers during care transitions. We conducted semi-structured interviews with caregivers (6 Spouses, 5 Children, 1 Friend) of hospitalized Veterans with dementia at the Michael E. DeBakey Veterans Affairs (VA) Medical Center. Interviews were recorded and transcribed verbatim. Rapid qualitative analysis using structured summaries and matrices were conducted. Although caregivers received information about the Veteran’s admitting medical condition, health professionals did not ask about dementia or provide dementia-related health information to the caregiver. Caregivers were willing to assist Veterans with activities of daily living in the hospital; however, caregivers were either prevented from assisting or did not receive training to assist Veterans safely. Caregivers desired information on how to get the Veteran “back on track” and address cognitive and functional declines experienced in the hospital. Although pre-hospital services and supports were easy to restart, caregivers needed earlier and more consistent communication regarding new services and supports starting after discharge, such as rehabilitation at skilled nursing facilities and respite care. Caregivers expressed a need for health professionals to help set “realistic expectations” for post-discharge care as some services and supports did not meet their expectations. Caregivers valued access to support groups for themselves but received inconsistent referrals to the VA Caregiver Support Program. These results will inform the adaptation of a dementia caregiver support intervention for caregivers of hospitalized Veterans living with dementia.

